# Regulatory Non-Coding RNAs in Familial Hypercholesterolemia, Theranostic Applications

**DOI:** 10.3389/fcell.2022.894800

**Published:** 2022-06-23

**Authors:** Hani Keshavarz Alikhani, Mahsa Pourhamzeh, Homeyra Seydi, Bahare Shokoohian, Nikoo Hossein-khannazer, Fatemeh Jamshidi-adegani, Sulaiman Al-Hashmi, Moustapha Hassan, Massoud Vosough

**Affiliations:** ^1^ Department of Regenerative Medicine, Cell Science Research Center, Royan Institute for Stem Cell Biology and Technology, ACECR, Tehran, Iran; ^2^ Gastroenterology and Liver Diseases Research Center, Research Institute for Gastroenterology and Liver Diseases, Shahid Beheshti University of Medical Sciences, Tehran, Iran; ^3^ Laboratory for Stem Cell and Regenerative Medicine, Natural and Medical Sciences Research Center, University of Nizwa, Nizwa, Oman; ^4^ Experimental Cancer Medicine, Institution for Laboratory Medicine, Karolinska Institute, Stockholm, Sweden

**Keywords:** familial hypercholesterolemia, low-density lipoprotein cholesterol, cardiovascular disease, advanced therapy, ncRNA-based therapy, ncRNA-based diagnosis

## Abstract

Familial hypercholesterolemia (FH) is a common monogenic disease which is associated with high serum levels of low-density lipoprotein cholesterol (LDL-C) and leads to atherosclerosis and cardiovascular disease (CVD). Early diagnosis and effective treatment strategy can significantly improve prognosis. Recently, non-coding RNAs (ncRNAs) have emerged as novel biomarkers for the diagnosis and innovative targets for therapeutics. Non-coding RNAs have essential roles in the regulation of LDL-C homeostasis, suggesting that manipulation and regulating ncRNAs could be a promising theranostic approach to ameliorate clinical complications of FH, particularly cardiovascular disease. In this review, we briefly discussed the mechanisms and pathophysiology of FH and novel therapeutic strategies for the treatment of FH. Moreover, the theranostic effects of different non-coding RNAs for the treatment and diagnosis of FH were highlighted. Finally, the advantages and disadvantages of ncRNA-based therapies vs. conventional therapies were discussed.

## 1 Background

Familial hypercholesterolemia (FH) is an autosomal co-dominant disorder that affects people of all ethnic backgrounds. The FH disease is characterized by abnormally high serum levels of low-density lipoprotein cholesterol (LDL-C), and if remains untreated, it can lead to atherosclerosis, cardiovascular diseases (CVDs), and even death (Chen, 2001; Hovingh et al., 2013). FH is a hereditary disorder in humans and is classified generally as heterozygous FH (HeFH) and Homozygous FH (HoFH) (Nordestgaard et al., 2013; Cuchel et al., 2014). The HeFH is associated with 2- to 3-folds higher cholesterol levels, but HoFH, the more aggressive and rare form of FH, is associated with 3- to 6-folds cholesterol levels higher than normal individuals. According to the previous research, the prevalence of HeFH is 1 case per 200–300 people (Goldstein et al., 1973), while the HoFH may affect up to 6 individuals per million (or 1 in 160,000–300,000) (Nordestgaard et al., 2013). Given the high prevalence of FH, feasibility in the measurement of LDL-C level, and considering the substantial socioeconomic burden of the disease, FH is severely underdiagnosed and undertreated (Nordestgaard et al., 2013; Watts et al., 2014; Gidding et al., 2015).

FH increases the risk of atherosclerosis and CVD, and approximately 2% of the patients with myocardial infarction at <50 years of age have a molecular diagnosis of FH (Do et al., 2015). Advanced age, elevated body mass index (BMI), elevated triglyceride levels, higher plasma LDL levels, reduced HDL cholesterol levels, type 2 diabetes, hypertension, and smoking are risk factors associated with CVD in HeFH, but these risk factors are not accompanied by HoFH (Wiegman et al., 2015; Pérez de Isla et al., 2016). Early diagnosis of the FH reduces the risk of CVD and is necessary for the use of cholesterol-reducing therapy and family screening to identify asymptomatic affected relatives (Umans-Eckenhausen et al., 2001). The first diagnostic approach is measuring LDL-Cholesterol level, which is clinically informative when it is > 4.9 mmol/L (190 mg/dl) in adults or 4.1 mmol/L (160 mg/dl) in children (Umans-Eckenhausen et al., 2001).

The gold standard for disease diagnosis is identifying a pathogenetic variant in the group of genes associated with FH. Because of high risk of CVD among individuals with pathogenetic variants, it could be argued that genetic testing should be performed in high risk individuals (Khera et al., 2016). Three important risk genes (*LDLR*, *APOB*, and *PCSK9*) are usually targeted for next-generation DNA sequencing. This method is the most commonly used one for molecular diagnosis, although bioinformatics analysis of whole-exome sequence data and the use of small non-coding RNAs (ncRNAs) focused on genes related to FH might be the methods of choice in the future (Iacocca and Hegele, 2017; Jiang et al., 2018).

## 2 Mechanisms and Pathophysiology of Familial Hypercholesterolemia

FH is associated with mutations in the genes that regulate the uptake of LDL particles from circulation. The HeFH and HoFH disorders are caused by autosomal dominant mutations in *LDLR* (LDL receptor), *APOB* (apolipoprotein B100), and *PCSK9* (proprotein convertase subtilisin/kexin type 9) genes (Nordestgaard et al., 2013; Gidding et al., 2015). Mutations in the *LDLR* can reduce the number of LDLR molecules or the LDLR activity in hepatocytes, lowering the LDL-C clearance from the plasma (Goldstein and Brown, 2009). More than 2900 different variants have been identified in the *LDLR* (Leigh et al., 2017) and more than 90% of these variants are likely to be pathogenic (Usifo et al., 2012). The apoB protein is the major lipoprotein in LDL-C and serves as a ligand for the LDL-receptor. Therefore, LDL particles with mutated and consequently defective apoB100 fail to bind to LDLR, and then the LDL-C particles accumulate in the plasma (Marais, 2004). PCSK9 activation leads to internalization and lysosomal degradation of LDL receptors, resulting in reduced LDL-C clearance from the circulation (Horton et al., 2009; Lakoski et al., 2009). Gain of function mutations or overexpression of the *PCSK9* gene is associated with rapid LDLR internalization and degradation and thus reduced the number of LDLR molecules in the cell membrane of hepatocytes (Goldstein et al., 1973). Some studies indicated that *LDLR* mutations are responsible for the highest mean levels of the LDL-C in most FH patients, whereas individuals with *APOB* or *PCSK9* mutations usually have somewhat lower levels (Nordestgaard et al., 2013; Cuchel et al., 2014). Interestingly, it should be noticed that the mutations of *LDLR*, *APOB,* and *PCSK9* are co-dominant, and on the other hand both mutated alleles are contributed to the disease phenotype; thus, gene-dosage and the association between the numbers of mutations can affect the intensity of the disease phenotype (Nordestgaard et al., 2013; Cuchel et al., 2014).

## 3 Management of Familial Hypercholesterolemia

The main goal of managing FH disease is to control the lipid levels in the patient’s sera. FH is being treated with different strategies, including lifestyle modifications and pharmacological therapy. The recommended goal for patients with FH is a 50% reduction of LDL-C in the sera (Defesche et al., 2017). According to the European Atherosclerosis Consensus panel, LDL-C values in HeFH should be < 3.5 mmol/L (135 mg/dl) in children and <2.5 mmol/L (100 mg/dl) in adults. The same LDL-C levels are also considered for HoFH patients, however, obtaining these LDL-C levels with currently available medications is challenging (Vallejo-Vaz et al., 2016). Furthermore, high-dose statins can reduce LDL-C levels by 10–25% in HeFH patients, while high-dose statins combined with ezetimibe can only reduce LDL-C levels by 20–30% in HeFH patients (Nordestgaard et al., 2013).

### 3.1 Novel Therapeutic Approaches

The US Food and Drug Administration (FDA) has approved four novel LDL-C-lowering drugs for HoFH and HeFH (Ajufo and Rader, 2018). Some monoclonal antibodies have been developed to inhibit enzymes or proteins involved in regulating LDL-C levels. Evolocumab, an anti-PCSK9 monoclonal antibody, was developed for HoFH patients in combination with other lipid-lowering therapies (Sabatine et al., 2017). Alirocumab is another anti-PCSK9 monoclonal antibody that has been developed for HeFH or clinical ASCVD patients (Robinson et al., 2015). Evinacumab, an inhibitor of ANGPTL3, is another monoclonal antibody that is in phase III clinical trial (Raal et al., 2020). Monoclonal antibodies also have some side effects; for example, infections of the upper respiratory tract, gastroenteritis, and nasopharyngitis, were the most common adverse effects of evolocumab (Ito and Watts, 2015).

Small molecules are new therapeutic agents that can inhibit specific targets in LDL-C metabolism. Due to ease of administration, low cost, long shelf life, and non-immunogenic response, small molecules are preferred drugs for the treatment of FH patients (Zhang et al., 2014a). Lomitapide (AEGR-733/BMS201038) is an orally administered small-molecule inhibitor of microsomal triglyceride transfer protein (MTP). It mediates the transfer of TG molecules to nascent apoB during the assembly of VLDL, and its inhibition decreases VLDL and eventually LDL assembly (Hooper et al., 2015). DS-9001a is a small biologic molecule (∼22 kDa) that inhibits PCSK9 binding to LDLR and attenuates LDLR degradation. Single intravenous injection of DS-9001a reduced LDL-C levels by ∼62%, and this effect was sustained for up to 21 days in the cynomolgus monkey (Masuda et al., 2018). 7030B-C5 is another small molecule that down-regulated *PCSK9* expression and increased the total cellular LDLR protein and mediated LDL-C uptake by HepG2 cells (Wang et al., 2020). Despite some advantages discussed, small molecules have several drawbacks, including low selectivity, non-tissue specific effects, and severe side effects, which limit their use as a drug (Miyosawa et al., 2015).

Mimetic peptides are also new therapeutic molecules designed to mimic peptides of a target protein. Many mimetic peptides that inhibit the PCSK9 have been developed. They mimic the EGF-A or EGF-AB binding domains of LDLR and bind to the catalytic domain of PCSK9, thereby reducing the chance of interaction between PCSK9 and LDLR (Craik et al., 2013). LDLR EGFA domain mimetic peptide was designed to inhibit PCSK9-mediated degradation of LDLR and increase the LDL uptake in a dose-dependent manner (Zhang et al., 2007). Moreover, the smaller mimetic peptide has been synthesized to inhibit the binding of PCSK9 to LDLR (Schroeder et al., 2014).

Vaccination therapy is an alternative strategy for the long-term reduction of LDL-C levels in FH patients. An anti-PCSK9 vaccine based on a designed peptide has been developed. This vaccine stimulates the immune system to produce antibodies against PCSK9 protein. Active vaccination offers some advantages over passive administration of PCSK9 monoclonal antibodies because active vaccination needs fewer injections, lower doses of drug administration, and reasonable cost. Importantly monoclonal antibodies may be neutralized by the host immune system (Nishikido and Ray, 2019). Landlinger *et al.* developed AT04A, a vaccine that mimics the fragments of a mature human PCSK9 protein conjugated to a foreign carrier protein (Keyhole limpet hemocyanin) for the management of FH disease (Landlinger et al., 2017). Mice vaccinated with AT04A have lower total cholesterol and LDL-C levels up to 30 and 50%, respectively (Galabova et al., 2014). A new method for developing innovative vaccines against self-antigens such as the PCSK9 is to use virus-like particles (VLPs) containing self-antigen on their surfaces. Crossey *et al.* designed a VLP-based vaccine targeting PCSK9, and they showed that the level of LDL-C reduced 30–40% in Macaques when combined with statins (Crossey et al., 2015). Furthermore, long-term efficacy is a crucial advantage of FH vaccination therapy, but potential adverse effects include antibody-dependent cell-mediated cytotoxicity or complement-dependent cytotoxicity, and non-specific cell destruction (Civeira and Jarauta, 2017).

The clustered regularly interspaced short palindromic repeats (CRISPR)/Cas9 system is a novel therapeutic approach for the treatment of monogenic inherited diseases (Zhao et al., 2020). CRISPR technology has some advantages including simple production, lower cost, and high efficiency (Jiang et al., 2018). In the FH, the CRISPR/Cas9 system can be employed to repair mutations in, for example, the *LDLR* gene or to knock out some genes related to diseases, such as *PCSK9*, *APOB*, and *ANGPTL3*. Omer *et al.* (2017) used the CRISPR/Cas9 technology to edit the 3 base-pair homozygous deletion in the exon 4 of *LDLR* gene in the HoFH patient-derived induced pluripotent stem cells (Omer et al., 2017). In the future, the CRISPR/Cas9 technology could be a potential therapeutic tool for the treatment of FH disease.

## 4 Non-Coding RNAs for the Treatment and Diagnosis of Familial Hypercholesterolemia and Other Dyslipidemia

### 4.1 Long Non-Coding RNAs (lncRNAs)

LncRNAs are defined as RNAs longer than 200 nucleotides (Huarte, 2015). The biogenesis of these RNAs is similar to mRNAs, and they are also processed by splicing, capping, polyadenylation, and editing (Klingenberg et al., 2017). lncRNAs lack significant open reading frames and thus have limited protein-coding potential. LncRNAs are transcribed by RNA polymerase (RNAP) II, and their expression is tightly regulated in various tissues (Vučićević et al., 2015; Shafabakhsh et al., 2021). Human GENCODE suggests more than 16,000 lncRNA genes in the human genome, but other databases estimated even more (Uszczynska-Ratajczak et al., 2018). LncRNAs are classified as long intergenic non-coding (linc)-RNAs, long non-coding antisense transcripts, long intronic ncRNAs, and non-overlapping antisense lncRNAs (Derrien et al., 2012). These RNAs have various regulatory functions at the chromatin level, including alternative splicing, cell differentiation, and cell cycle regulation (Meng et al., 2020a). Also, lncRNAs have regulatory roles in many diseases (Meng et al., 2020a). Recently, it has been demonstrated that some lncRNAs such as MeXis, LeXis, and ApoA4-AS can control lipid metabolism in different tissues (Zhang et al., 2019).

#### 4.1.1 Mechanism of Biogenesis

The biogenesis of lncRNAs is a multi-step process, and different mechanisms are involved, including RNaseP digestion (for the generation of mature ends), snoRNP complex formation (for insertion of a cap to the end of lncRNAs), and formation of circular structures (Chen, 2016; Dashti et al., 2021). Previously, scientists thought that the transcription and processing of lncRNAs were similar to mRNAs, but recently some studies revealed distinct features in transcription, processing, export, and turnover of lncRNAs (Statello et al., 2021). The co-transcriptionally splicing of lncRNAs is weakly performed in the nucleus and the transcription termination is independent of polyadenylation signals (Schlackow et al., 2017). Therefore, the lncRNAs accumulate in the nucleus and then are degraded by RNA exosomes (Schlackow et al., 2017). In comparison to mRNAs, lncRNA genes are less evolutionarily conserved, contain fewer exons, and are less abundantly expressed (Guo et al., 2020). The low expression of lncRNAs is probably dependent on repressive histone modifications at their promoters (Melé et al., 2017). Most lncRNAs are found in the nucleus due to the high levels of U1 small nuclear RNA binding sites on lncRNA (Yin et al., 2020), the loss of function of the Pol II-associated elongation factor SPT6 (Nojima et al., 2018), and differential expression of specific splicing regulators (Melé et al., 2017). Nevertheless, certain lncRNAs are exported to the cytosol with the same processing and export pathways as mRNAs (Statello et al., 2021). The nuclear RNA export factor 1 (NXF1) pathway is responsible for exporting long and A/U-rich transcripts of lncRNAs with one or two exons (Zuckerman et al., 2020). They undergo specific sorting processes and each lncRNAs distributed in a specific organelle or cytoplasm (Statello et al., 2021).

Circular RNAs (circRNAs) are single stranded RNAs that are covalently looped. There are three types of circRNAs based on different circularizing mechanisms, including exonic circRNAs, intronic circRNAs, and intron-retained circRNAs (Chen, 2016). Usually, circRNAs are originated from exons, but others are also generated from 3´UTR, 5´UTR, introns, intergenic regions, and antisense RNAs (Li et al., 2015a). These RNAs are less efficiently transcribed than linear RNAs, but they are stable, and thus their number in the cells are still substantial (Qu et al., 2015). The mechanism of biogenesis of circRNAs is not completely clear, but the back-splicing mechanism depends on complementary intron matches is involved (Zhang et al., 2014b). To generate circRNAs, the back-splicing occurs in reversed orientation that connects a downstream 3´ splice site to an upstream 5´ splice site (Zhang et al., 2016). Generation of intronic circRNA depends on the splicing mechanism, and the GU-rich sequences near the 5´ splice site and the C-rich sequences near the branch point are needed. During the back-splicing process, the two segments bind to form the circle and then the exonic and intronic sequences are cut out by the spliceosome. After all, the introns are pieced together to form mature circRNAs (Talhouarne and Gall, 2014; Chen, 2016).

#### 4.1.2 Mechanism of Action

LncRNAs have various regulatory functions. At the chromatin level, they are involved in dosage compensation, genomic imprinting, chromatin modification, and remodeling (Kanduri, 2016). At the transcription level, lncRNAs regulate alternative splicing, change the activity of transcriptional factors, and modify the activity of RNAP II (Zhao et al., 2008). LncRNAs also have a role in regulating cell differentiation and cell cycle, as well as the incidence of many diseases (Kitagawa et al., 2013; Fatica and Bozzoni, 2014; Yang et al., 2014). The cis and trans-acting modulator function of lncRNAs is crucial for the expression of many protein-coding genes (Mao et al., 2011). Also, these RNAs recruit the chromatin-remodeling complex to a specific locus of the chromatin and help regulate epigenetic changes in the genome (Bhat et al., 2016). Some of the well-known lncRNAs involved in epigenetic changes are ANRIL, XIST, HOTAIR, and KCNQ1OT1 (Bhat et al., 2016). LncRNAs interact with RNA-binding factors such as hnRNPs to either promote or suppress gene transcription. These RNAs also recruit transcription factors to the adjacent target genes to enhance their transcription (Bhat et al., 2016). Some lncRNAs regulate transcription by binding to regulatory proteins, RNA, or DNA molecules and sequestering them from their target site (Chowdhary et al., 2021). Some lncRNAs can identify complementary sequences in target RNAs, enabling them to participate in post-transcriptional regulation. Particularly, some lncRNAs can act as a sponge and bind to miRNAs and prevent their interaction with their target mRNAs (Salmena et al., 2011). Interestingly, the miRNA Response Element (MRE) located in the 3′ end of most lncRNAs is in charge of capturing miRNAs, because this reign is complementary with the Ago binding sites which were presented in the most of miRNAs (Jalali et al., 2013). LncRNAs are also involved in the maturation, transportation, degradation, and stability of mRNAs. For example, the NAT lncRNA has an essential role in the regulation of mRNA dynamics (Kim et al., 2020).

circRNAs are important regulatory elements that regulate at both transcription and post-transcription levels. They act as miRNA sponges and compete with endogenous RNAs (ceRNAs). As ceRNAs, they can compete for miRNA-binding sites and regulate mRNA expression (Memczak et al., 2013). Also, circRNAs can bind to RBPs and regulate the transcription, and in some cases translation (Altesha et al., 2019). It was shown that a group of regulatory circRNAs, i.e., exon-intron circRNAs (EIciRNAs), play an important role in gene expression and transcription (Li et al., 2015b). The circRNAs encapsulated into exosomes have a role in cellular communications and physiological and pathological conditions (Rayner and Hennessy, 2013).

#### 4.1.3 Theranostic Application of lncRNAs in Dyslipidemia and Familial Hypercholesterolemia

lncRNAs are involved in cholesterol homeostasis and lipid-related diseases such as cardiovascular diseases (Ou et al., 2020). Next-generation sequencing is one of the best techniques to recognize novel lncRNAs related to FH (Chowdhary et al., 2021). Other methods such as microarray and RNA-Seq technologies are used to investigate the effects of lncRNAs on lipid metabolisms (Chowdhary et al., 2021). Computational tools combined with experimental techniques are promising approaches for detecting novel lncRNAs and also for the recognition of their role in lipid metabolisms and related diseases (Jalali et al., 2015).

Many circRNAs have been identified that have role in FH, CHD, atherosclerosis, myocardial fibrosis (MF), and other cardiovascular diseases (Gabriel et al., 2020; Lim et al., 2020). A study conducted by Sun, Y. *et al.* reported that 447 circRNAs were upregulated and 219 circRNAs were downregulated in FH (Sun et al., 2020). circHRCR is another circRNA which was reported as an endogenous miR-223 sponge and has a role in the inhibition of cardiac hypertrophy and HF (Wang et al., 2016).

**FIGURE 1:**
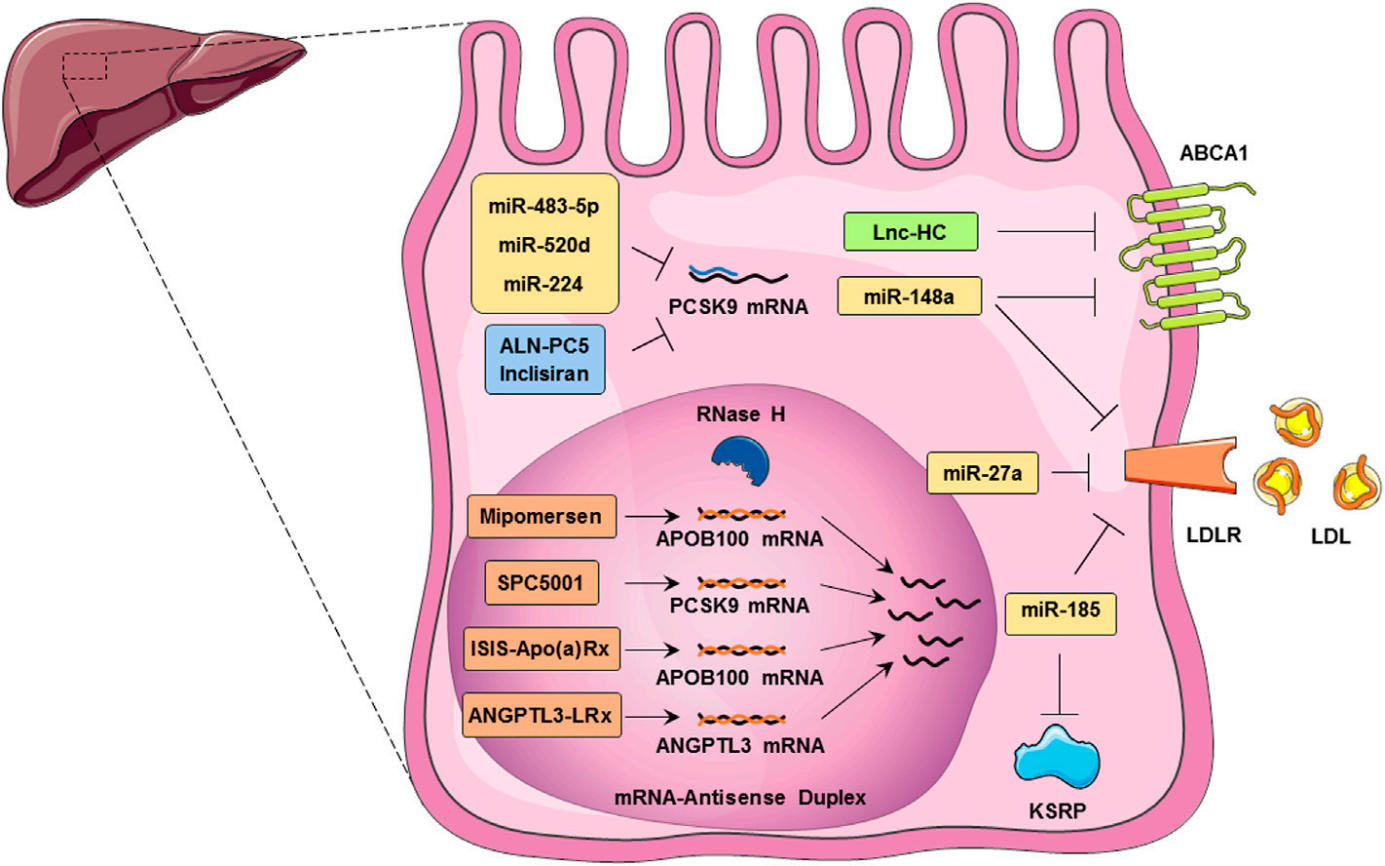
Noncoding RNAs regulate cholesterol homeostasis in hepatocytes. These RNAs, including, LncRNAs, miRNAs, ASOs, and siRNAs, control cholesterol accumulation, cholesterol efflux, cholesterol biosynthesis, and cholesterol metabolism. ABCA1 expression is regulated by both Lnc-HC and miR-148a in hepatocytes which leads to cholesterol efflux inhibition. MicroRNAs such as miR-483-5p, miR-520d, and miR-224 inhibit PCSK9 translation. miR-27a and miR-185 are involved in the inhibition of LDLR translation and thus reduce the LDL uptake by hepatocytes. Mipomersen, ISIS-Apo(a)Rx, and ANGPTL3-LRx are antisense oligonucleotides that regulate the APOB100 mRNA. SPC5001 induces the degradation of PCSK9 mRNA in the nucleus of the hepatocytes. Inclisiran, an approved siRNA, and ALN-PC5 inhibit the translation of PCSK9 in the cytoplasm of hepatocyte and hence LDLR internalization.

As a therapeutic approach, lncRNAs may serve as key regulators of lipid homeostasis. Here we briefly highlighted various lncRNAs involved in cholesterol metabolism and their potential therapeutic applications (Figure 1; Table 1).

**TABLE 1:** Application of different non-coding RNAs in FH.

Types of non-coding RNAs	RNAs	Functions	References
Long non-coding RNAs	MIAT	Down-regulation leads to lowering atherosclerotic plaques and lipid profiles	Sun et al. (2019)
	GAS5	Down-regulation repress the progression of lipid accumulation and atherosclerosis	Shen et al. (2019)
	Rp5-833A20.1	Down-regulation may reduce the amount of LDL-c and VLDL-c in sera	Hu et al. (2015)
	LeXis	Up-regulation reduce the total serum cholesterol levels	Muret et al. (2019)
	Lnc-HC	Down-regulation improves the total cholesterol, TG, and HDL-cholesterol levels	Zhao et al. (2017)
	DYNLRB2	Down-regulation reduce the circulating lipids and alleviate atherosclerotic symptoms	Zhao et al. (2017); and Zhang et al. (2019)
Small interfering RNAs	ALN-PCS	Reduces the PCSK9 mRNA and LDL-C levels	Graham et al. (2007)
	Inclisiran	Reduces the PCSK9 mRNA and LDL-C levels	Nishikido et al. (2019)
	SNALP-siRNA	Reduces the apoB mRNA and LDL-C levels	Zimmermann et al. (2006)
Antisense oligonucleotides	2’-O-methoxyethyl ASO	Reduces the PCSK9 mRNA and LDL-C levels	Graham et al. (2007)
	SPC5001	Reduces the PCSK9 mRNA and LDL-C levels	Gupta et al. (2010)
	Mipomersen	Reduces the apoB mRNA and LDL-C levels	Gaudet et al. (2015)
	ISIS-APO(a)Rx	Targeted the Lp(a) protein level to reduce the early onset and severity of coronary artery disease (CAD)	Li et al. (2017)
	ANGPTL3-LRx	Decreases ANGPTL3 protein expression, TGs, LDL-C, VLDL, apoB, and apoC-III levels	Graham et al. (2017)
MicroRNAs	miR-148a	Affects HDL-C levels by negative regulation of LDLR mRNA translation	Goedeke et al. (2015)
	miR-27a	Inhibits the expression of LDLR and LDLR-associated factors, including LRP6, LDLRAP1	Alvarez et al. (2015)
	miR-185	Down-regulating the RNA-binding protein KH-type splicing regulatory protein as well as directly targeting LDLR (KSRP).	Jiang et al. (2015)
	miR-221	Reduces the PCSK9 mRNA and LDL-C levels	Naeli et al. (2017)
	miR-224	Reduces the PCSK9 mRNA and LDL-C levels	Naeli et al. (2017)
	miR-191	Reduces the PCSK9 mRNA and LDL-C levels	Naeli et al. (2017)
	miR-483-5p	Targeting the 3′-UTR of PCSK9 mRNA and decreases the circulating LDL-C	Dong et al. (2020)
	miR-34a	Reduces the apoB mRNA and LDL-C levels	Xu et al. (2015)
	miR-224	Reduces the PCSK9 mRNA and LDL-C levels	Salerno et al. (2020)
	miR-520d	Reduces the PCSK9 mRNA and LDL-C levels	Salerno et al. (2020)
	miR-30c	Reduces the microsomal triglyceride transfer protein (MTP) mRNA levels	Irani et al. (2018)
	miR-133a	A diagnostic marker for cardiovascular diseases and atherosclerosis in FH patients	Escate et al. (2021)
	miR-200c	A diagnostic marker for FH	Escate et al. (2021)
	miR-30a/b	Used as a diagnostic marker for cardiovascular disease	D’Agostino et al. (2017)
	miR-223	Used as a diagnostic marker for FH disease	Vickers et al. (2011)
	miR-105	Used as a diagnostic marker for FH disease	Vickers et al. (2011)
	miR-106a	Used as a diagnostic marker for FH disease	Vickers et al. (2011)
	miR-486	Used as a diagnostic marker for FH disease	Scicali et al. (2019)
	miR-92a	Used as a diagnostic marker for FH disease	Scicali et al. (2019)

##### 4.1.3.1 Myocardial Infarction-Associated Transcript

**FIGURE 2:**
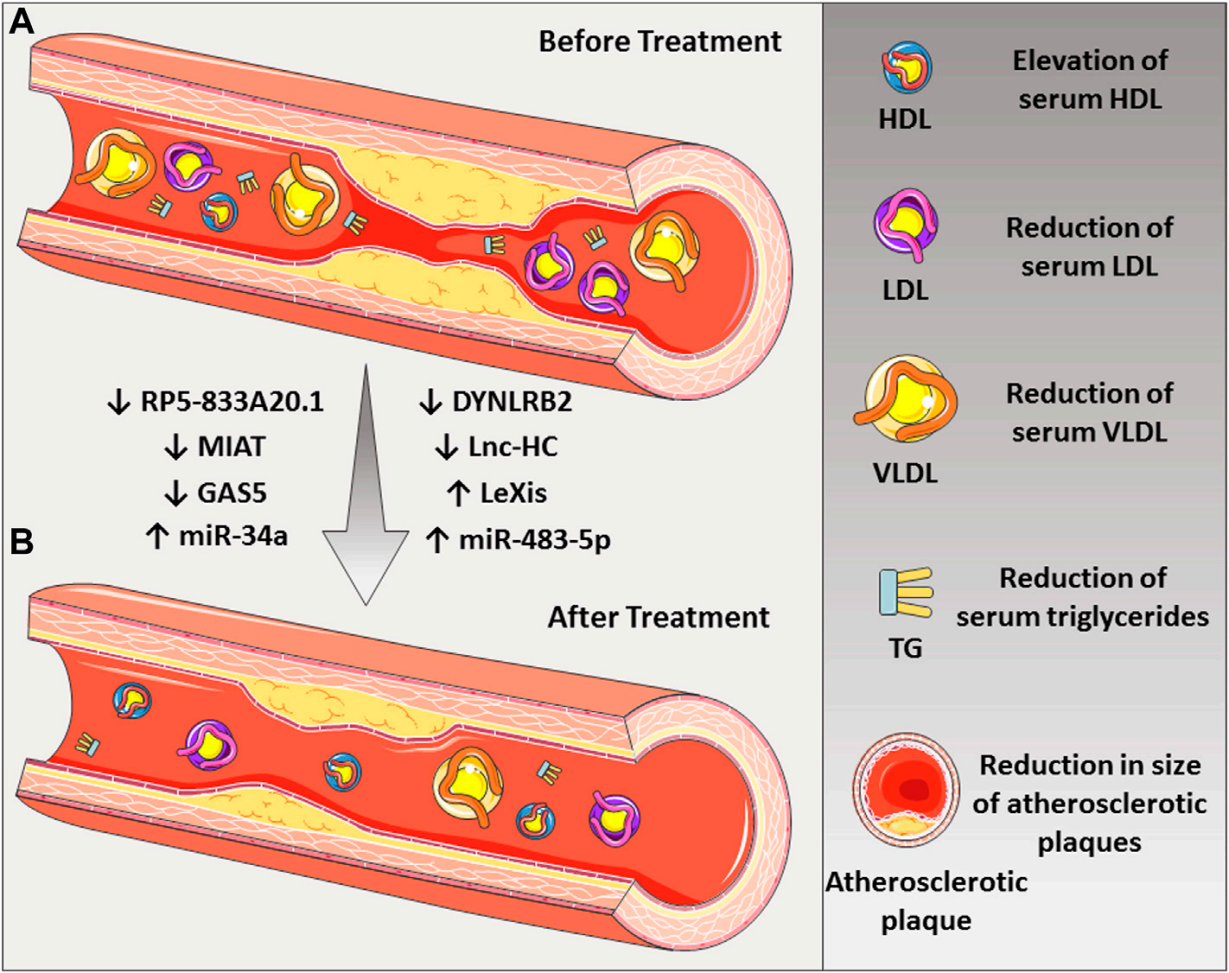
The roles of various noncoding RNAs in the treatment of atherosclerotic disease. **(A)** Down-regulation of some lncRNAs such as MIAT, GAS5, RP5-833A20.1, DYNLRB2, and Lnc-HC and **(B)** up-regulation of LeXis lncRNA could lead to the reduction of LDL-C and atherosclerotic plaque. Also, up-regulation of miR-34a and miR-483-5p could be beneficial in the treatment of the disease.

*MIAT* is a long non-coding RNA located on chromosome 22q12.1 and regulates the expression of genes at both transcriptional and post-transcriptional levels (Ghafouri-Fard et al., 2021). Many processes in target cells are influenced by the MIAT lncRNA, such as cell proliferation and apoptosis, cell cycle progression and migration, as well as lipid metabolism (Da et al., 2020). It has been reported that MIAT can promote the production of inflammatory factors such as IL-1β, IL-6, and TNF-α in atherosclerotic mice model *via* the PI3K/Akt signaling pathway (Sun et al., 2019). The PI3K/Akt signaling pathway activation can reduce reactive oxygen species and lipid deposits, which could suppress the formation of the atherosclerotic plaques (Luo et al., 2015). MIAT up-regulation activates the PI3K/Akt signaling pathway and thus aggravates the development of atherosclerosis in the mice model (Sun et al., 2019). Also, it is worth mentioning that MIAT is increased in patients with high Lp(a) (Fasolo et al., 2021). Therefore, down-regulation of this lncRNA can be a potential therapeutic target for lowering atherosclerotic plaques and lipid profiles (Figure 2).

##### 4.1.3.2 Growth Arrest-specific 5

*GAS5* gene comprises 12 exons, located at 1q25.1. It encodes long noncoding RNA (Pickard and Williams, 2015). *GAS5* is up-regulated in atherosclerosis plaque as compared with normal people (Chen et al., 2016a). LncRNA GAS5 binds to the zeste homolog 2 (EZH2) enhancer element, inhibiting the expression of the *ABCA1* gene in THP-1 macrophages. In *APOE*-deficient mice, inhibiting *ABCA1* reduces cholesterol efflux as well as the progression of atherosclerosis (Meng et al., 2020b). In the atherosclerotic and apoE^−/−^ mice model, up-regulation of *GAS5* increases the levels of LDL-C, total cholesterol, and aortic plaques (Meng et al., 2020b). In contrast, *GAS5* silencing can repress the progression of lipid accumulation and atherosclerosis (Shen et al., 2019), suggesting that *GAS5* targeting might be a promising approach for atherosclerosis (Figure 2).

##### 4.1.3.3 RP5-833A20.1

The *RP5-833A20.1* lncRNA is located in an opposite transcriptional direction of the Nuclear Factor IA (*NFIA*) gene (Kang et al., 2016). In macrophage-derived foam cells, up-regulation of the RP5-833A20.1 lncRNA results in down-regulation of the *NFIA* gene. It was shown that overexpression of the *NFIA* gene is associated with increased HDL-C circulation, decreased LDL-C cholesterol, and VLDL-C circulation in apoE^−/−^ mice (Hu et al., 2015). Therefore, inhibition of RP5-833A20.1 expression may represent a therapeutic target to reduce LDL-c and VLDL-c in sera (Ye et al., 2021).

##### 4.1.3.4 Liver-Expressed LXR-Induced Sequence (LeXis)

As a regulator of hepatic sterol content and serum cholesterol levels, LeXis lncRNA orchestrates the crosstalk between liver X receptor and SREBP transcription factors (Sallam et al., 2016). It was demonstrated that LeXis decrease cholesterol biosynthesis via binding to RALY (a heterogeneous ribonucleoprotein) and enhance cholesterol efflux in the liver (Muret et al., 2019). While LeXis lncRNA regulates RALY, it interacts with DNA as a transcription factor and inhibits the genes involved in the synthesis of cholesterol. It was shown that adenoviral administration of LeXis in mice results in reducing the total serum cholesterol levels (Sallam et al., 2015; Zhao et al., 2017).

##### 4.1.3.5 Lnc-HC

Lnc-HC regulates hepatocyte cholesterol catabolism and is found in the liver and adipocytes. The lnc-HC forms the lncRNA-protein complex with hnRNPA2B1 and as a co-regulator binds to the mRNAs encoding Cyp7a1 and Abca1 and decreases their stability. Cyp7a1 and Abca1 are critical enzymes in cholesterol catabolism (Lan et al., 2016; Lan et al., 2019). Inhibition of Lnc-HC significantly increased the expression of *CYP7a1* and *ABCA1* in hepatocytes. It is now clear that lnc-HC knockdown improves the total cholesterol, TG, and HDL-cholesterol levels in rats (Zhao et al., 2017).

##### 4.1.3.6 DYNLRB2

DYNLRB2 is a long intergenic non-coding RNA expressed in human macrophages in response to oxidized-LDL (ox-LDL) (Hu et al., 2014). In macrophage foam cells, overexpression of the DYNLRB2 lncRNA promotes the expression of the ABCA1 and G protein-coupled receptor 119 (*GPR119*) genes, which increase cholesterol efflux and decrease neutral lipid accumulation. Down-regulation of DYNLRB2 in mice decreased circulating lipids and alleviate atherosclerotic symptoms (Zhao et al., 2017; Zhang et al., 2019).

### 4.2 MicroRNAs

#### 4.2.1 Mechanism of Biogenesis

As small but vitally regulatory modules, micro-RNAs that vary in length from 18 to 24 nucleotides belong to the ncRNAs class (Ergin and Çetinkaya, 2022). These molecules, which are dysregulated in many diseases and cancers, influence gene expression in the post-translational phases of numerous processes, including cell growth and development (Annese et al., 2020). In the first step, miRNA synthesis starts in the nucleus, and other consecutive steps occur in the cytoplasm. This pathway is multistep: in step 1, RNAP II and III execute transcription of miRNA genes that are generally assigned to miRNA-specific genes (Pourhanifeh et al., 2020; Dashti et al., 2021).

The hairpin primary miRNA sequence (pri-miRNA) produced at this stage is capable of producing one miRNA or, in some cases, more than one miRNA (Lin and Gregory, 2015). Then, in step 2, pri-miRNA cleaved to pre-miRNA by Drosha and DiGeorge critical region 8 (DGCR8). In step 3, Exportin 5 (Exp5) exports the produced pre-miRNA into the cytoplasm (Lee and Shin, 2018; Treiber et al., 2019). In the fourth step, the Dicer enzyme cleaves the pre-miRNA to miRNA duplex in the cytoplasm. Finally, one strand (guide strand) of duplex miRNA is linked to the Argonaute (Ago) protein to create the RNA-induced silencing complex (RISC), and miRNA was activated (Detassis et al., 2017). The nomenclature of the miRNA strand is due to the origination of 3′-end or 5′-end of pre-miRNA source (Zaporozhchenko et al., 2020).

In addition to canonical rout, there are some atypical miRNA biogenesis pathways that can be relevant to CVD. One class of miRNAs biosynthesized in these non-canonical pathways are miRtrons that are derived from introns. Due to the short length of a hairpin structure, after splicing in the nucleus and export to the cytoplasm, processes of pre-miRNA are done by Dicer instead of Drosha and DGCR8 (Lopez-Pedrera et al., 2020). Despite Dicer dependent-pathway, some miRNA are synthesized in a Dicer-independent manner. For instance, because of miR-451 precursor’s cleavage by Drosha/Dgcr8, the product is not long enough to cleave by Dicer. Hence, Pre-miR-451 was cleaved by unknown enzymes and then loaded into the Ago-RISC complex (Natarelli and Weber, 2022). Furthermore, there are some miRNAs localized in the nucleus through specific nucleotide motifs. These Atypical miRNAs such as miR-320, Let- 7d, and Let-7i can regulate the expression of genes at epigenetic levels. Besides, miR-126-5p and miR-133a as nuclear miRNAs implicates in atherosclerosis and CVD (Di Mauro et al., 2019; Santovito et al., 2020a). The microRNA duplexes with both active strands are another group of non-canonical miRNA. Conventionally one strand of cleaved miRNA loaded to RISC was named the guide strand, and another passenger strand was degraded. Interestingly, in microRNA duplexes such as miR-126, which are related to cardiovascular diseases, both guide and passenger strands are functional (Santovito and Weber, 2022).

#### 4.2.2 Mechanism of Action

microRNAs as critical gene regulators can affect the function and developmental processes of human cells. miRNAs negatively regulate target genes expression. By cleaving target mRNA in the RISC complex (miRISC), it completely stops mRNA translation (Zhang et al., 2020). The GW182 factor, as well as its human homologs TNRC6A, B, and C, play an important role in the RISC function (Zaporozhchenko et al., 2020). Usually, target mRNA through complement nucleotides in its 3′ UTR region can interact with the seed sequence of miRNA (nucleotides 2-8 from the 5´-end of the miRNA). Translation of target mRNA is inhibited and then degraded by entirely complementary binding of miRNA and a specific region of 3′ UTR mRNA (perfect complementarity) (Annese et al., 2020; O'Brien et al., 2018). Additionally, especially in mammals, translational suppression is linked to partial complementary base pairing including mismatches and bulges (Lee et al., 2009; Ekimler and Sahin, 2014).

#### 4.2.3 Role of miRNAs in Treatment of Familial Hypercholesterolemia

Currently, FH is treated with conventional medicines that lower LDL-C levels that have risen due to mutations in the LDLR gene or associated proteins (Pedro-Botet et al., 2021). However, the results suggest that using these medicines does not result in an acceptable level of LDL-C (Momtazi et al., 2018). Hence, it seems that regulating FH associated genes that participate in cholesterol hemostasis through miRNA could be a promising therapy (Ahangari et al., 2018). On the one hand, some silencing miRNA can attenuate the FH and related cardiovascular signs. For instance, in one way, miR-148a affects LDL-C levels by down-regulation of LDLR mRNA translation. In addition, the down-regulation of ABCA1 mRNA by miR-148a can lower the cholesterol (that affects circulating HDL) efflux to apoA1 in Huh7 cells. On the other hand, *in vivo* inhibition of mir-148a reverses the negative regulatory effect on LDLR and ABCA1 mRNA (Goedeke et al., 2015). Besides, ABCA1 and ABCG1 genes are targeted by miR-33, a well-known miRNA involved in cholesterol efflux. Inhibition of this miRNA influences cholesterol efflux and promotes HDL circulating levels (Rayner et al., 2010; Das et al., 2018). Despite that statins are being considered as an LDL-C reducing drug, it is worth mentioning that they can impact miR-33 expression. The result showed that treatment with atorvastatin and pitavastatin could lead to miR-33 overexpression which can cause ABCA1 down-regulation and lowering cholesterol efflux in Macrophages in a dose-dependent manner (Chen et al., 2016b; Tsilimigras et al., 2021). Ubilla and colleagues compared the levels of circulating miRNAs in hypercholesterolemia (HC) patients treated with atorvastatin and in the control group. The findings show that atorvastatin treatment (20 mg/day) causes a significant increase in miRNA-33b-5p levels in HC patients (Ubilla et al., 2021). In contrast, Santovito *et al.* confirmed that high doses of rosuvastatin (40 mg) up-regulate the ABCA1 protein in macrophages through down-regulation of miR-33b-5p in HC patients (Santovito et al., 2020b). miR-27a regulates cholesterol homeostasis both directly and indirectly. miR-27a inhibits the expression of LDLR and LDLR-associated factors, including LRP6, LDLRAP1, which are involved in LDL endocytosis, while promoting the expression of PCSK9 (Alvarez et al., 2015). miR-185 regulates LDL absorption via down-regulating the RNA-binding protein KH-type splicing regulatory protein (KSRP) as well as directly targeting LDLR. As a result, inhibiting miR-185 may be a potential method for treating atherosclerosis (Jiang et al., 2015). Overexpression of other miRNAs, on the other hand, is possibly effective in FH treatment. According to the findings, there is a negative association between the expression levels of miR-221, miR-224, and miR-191 with PCSK9 in hepatoma cells. The aforementioned miRNAs have been connected to hypercholesterolemia because they can interact directly with PCSK9's mRNA (Naeli et al., 2017). Intracellular PCSK9 can cause LDL-R breakdown in the lysosome and reduce LDL-C uptake. In humans, there is an inverse association between miR-483-5p levels and circulating LDL-C. Up-regulation of miR-483-5p resulted in a decrease in circulating LDL-C and increased LDL-R presence on the cell surface via robust targeting of the 3′-UTR of PCSK9 mRNA (Dong et al., 2020). ApoB protein was considerably down-regulated in HepG2 cells treated with miR-34a. miR-34a, a lipid metabolism regulator, modulates hepatic *HNF4a* expression both *in vivo* and *in vitro*. The results showed that miR-34a could reduce VLDL secretion while increasing hypolipidemia (Xu et al., 2015). Similar research has found that miR-224 and miR-520d, and miR-30c can inhibit PCSK9 and microsomal triglyceride transfer protein (MTP), respectively. These miRNAs can help with LDL clearance, lowering plasma LDL-C, and lessening the effects of hypercholesterolemia (Irani et al., 2018; Salerno et al., 2020) (Figure 1; Table 1).

#### 4.2.4 Role of miRNAs in Diagnosis of Familial Hypercholesterolemia

miRNAs are intriguing new biomarkers that are crucial not only in FH therapeutic research but also in the diagnosis and prognosis of the disease. A cohort study was used to examine miRNAs in FH patients who had a cardiovascular event (FH-CVE) and FH patients who did not have a cardiovascular incident (FH-nCVE). miR-133a was a marker that was elevated in the FH-CVE population. Because miR-133a is linked to inflammatory cytokines, this could be used as a diagnostic marker for cardiovascular and atherosclerosis in FH patients (Escate et al., 2021). Notably, the increased level of the miR-133a was found in vulnerable human atherosclerotic plaques in dyslipidemic patients (Cipollone et al., 2011). Furthermore, researchers discovered that the level of miR-200C in plasma from children with FH was much higher than in healthy subjects. This miRNA's expression corresponds to the level of miR-30a/b, and it appears to be used as a diagnostic marker for cardiovascular disease susceptibility (D’Agostino et al., 2017). Many studies have tried to show HDL-miRNAs as disease biomarkers due to their accessibility in plasma (Vickers and Michell, 2021). Previously, FH patients and healthy people were tested for the whole HDL micro-transcriptome. The data showed a significant difference in miR-223, miR-105, and miR-106a expression between the two groups. Furthermore, miR-223 has a 3780.6-fold change in the FH population, suggesting that it has an impact on the gene expression profile of HDL-recipient cells (Vickers et al., 2011). Comparing HDL miRNA panel expression in the LDLR-null group to the LDLR-defective group, the result showed high expression of miR-486 and miR-92a in the former (Scicali et al., 2019) and these up-regulating miRNAs in HDL are related to CAD (Niculescu et al., 2015).

### 4.3 Small Interfering RNAs

siRNAs (20–30 nucleotide-long) are synthesized artificially and employed as novel therapeutic agents. These RNAs can bind to target mRNAs and help to silence disease-causing genes (Zimmermann et al., 2006). As compared with small molecules and monoclonal antibodies, siRNAs have some advantages. Small molecules and monoclonal antibodies need to bind to the spatial conformation of target molecules (for example, proteins), but siRNAs can bind to the target mRNA by just Watson–Crick base pairing pattern. As a result of base-pairing, siRNAs can target many mRNAs, enabling the treatment of a wide range of disorders (Watanabe et al., 2009). siRNAs have some disadvantages; for example, their delivery to the targeted tissues and their stability are still facing challenges (Rinaldi and Wood, 2018). Recently some siRNAs have been developed to treat FH by targeting various genes in this pathway.

#### 4.3.1 Synthesis and Mechanism of Action of Small Interfering RNAs

Many siRNAs are developed for targeting various genes in different diseases. siRNAs mimic endogenous miRNA duplexes to silence gene expression. Commercially, siRNAs are synthesized via the solid-phase synthesis method (Crooke, 2017). siRNAs can be ordered as pre-formed duplexes or single-stranded RNA. *In-vitro* transcription of siRNAs and shRNAs is another way for synthesizing siRNAs, but due to the immuno-stimulatory effects, the 5′ triphosphate at the end of siRNA must be removed using phosphatases (Salomon et al., 2015). Some new siRNAs are chemically modified by substituting the 2′-OH with a 2′-O-methyl or 2′-methoxyethyl group (Crooke et al., 2007). Moreover, some siRNAs can be modified by substitution of specific nucleotides; for example, locked, unlocked, and glycol nucleotides can be inserted into siRNAs. These modifications can be useful for suppressing the immuno-stimulatory effects of siRNAs and enhancing their activity, specificity, and, reducing their off-target-induced toxicity (Rozema, 2017).

Dicer cleaves the long dsRNA in the target cell to form the siRNA duplex (Dias and Stein, 2002). Dicer is a member of the RNase III family that processes small RNA molecules to produce functional RNAs. The duplex siRNA has a passenger or sense strand and a guide or antisense strand. In the first step, RNA forms a pre-RISC complex with Dicer, which is then cleaved by this enzyme. Two proteins, the TRBP (HIV trans-activating response RNA-binding protein) and PACT (protein activator of PKR) are involved in capturing the RNA molecule for further processing (Watanabe et al., 2006). One of the captured RNA strands is then selected as the guide strand and binds to the Argonaute (AGO) protein to form the RNA-induced silencing complex (RISC) complex. In the RISC complex, the duplex RNA molecule is unwound and the passenger strand is degraded. The thermodynamic asymmetry properties of the end of RNA strands are the main factor in determining the guide or passenger strands. The guide strand usually has no stable 5’ end compared to the passenger strand (Geary et al., 2001). Then, the RISC complex was activated and recognized a specific target site in the single-stranded guide RNA molecule. Therefore, the RISC-siRNA complex is ready to bind to the particular sequence of the target RNA molecule and either inhibit the translation of the target messenger RNA or degrade the complementary RNA (Hamm et al., 2010).

#### 4.3.2 Small Interfering RNAs in Familial Hypercholesterolemia

The ALN-PCS, which targets the PCSK9 mRNA, is one of the best examples of modified siRNAs used to treat FH disease. Treatment with this molecule reduces PCSK9 mRNA and LDL-C levels (Graham et al., 2007). Intravenous infusion of ALN-PCS in healthy volunteers reduced the PCSK9 mRNA level by 70% and the LDL-C levels by 40% after 3 days of infusion (Hudziak et al., 1996). Inclisiran (ALNPCSsc: ALN-60,212) is an improved version of ALN-PCS composed of nucleotides with 2′-deoxy, 2′-fluoro, and 2′-O-methyl groups. N-acetylgalactosamine (GalNAc) is conjugated to the 3′-end of Inclisiran to target the drug to the asialoglycoprotein receptors in the liver (Nishikido and Ray, 2019). This receptor is highly expressed in the hepatocytes and is a promising option for the effective uptake of inclisiran as well as a protective mechanism against off-target effects (Nishikido and Ray, 2019). Therefore, 24 h after subcutaneous injection, the drug's plasma level had drastically reduced to undetectable levels (Gupta et al., 2010). The results of the phase III study of inclisiran showed that twice administration (yearly) accompanied by statin therapy is a safe, effective, and well-tolerated regiment to lower LDL-C in adults with HeFH (van Poelgeest et al., 2015) (Figure 1).

In a study, 84 siRNA molecules specific to human and mouse ApoB mRNA were designed and synthesized using bioinformatic methods. The qRT–PCR and ELISA results showed that five siRNAs could reduce both mRNA and protein levels by 70% (van Poelgeest et al., 2013). In another study, ApoB-specific siRNA was encapsulated in Stable Nucleic Acid Lipid Particle (SNALP) and administered by intravenous injection. Results showed that single-dose injection reduced the ApoB mRNA expression in the liver as measured by stem-loop RT-qPCR. Further experiments showed that the siRNA encapsulated in SNALP significantly reduced the serum levels of cholesterol and LDL in LDL receptor knockout mice (Zimmermann et al., 2006). The combination of ApoB-specific siRNA and dendritic poly (L-lysine) administered intravenously suppressed *APOB* expression in C57BL/6 mice without causing hepatotoxicity. Results demonstrated that the level of LDL-C reduced significantly (Watanabe et al., 2009; Table 1).

### 4.4 Antisense Oligonucleotides

ASOs are synthetic small non-coding RNA molecules that influence RNA processing and protein expression and are used in the clinic as promising alternative to target specific genes (Rinaldi and Wood, 2018). Four mechanisms for the ASO-mediated down-regulation of target genes include, RNase H endonuclease mediated cleavage of the RNA-DNA hetero-duplex, inhibition of 5′ cap formation, alteration of splicing process (splice-switching), and steric hindrance of ribosomal activity (Bennett et al., 2017; Crooke, 2017). The RNase H endonuclease mechanism was described well and had three phases: pre-hybridization, hybridization, and post-hybridization. In the pre-hybridization step, the ASO enters the target cell and achieves effective concentration at the site of interest. Then, the ASO aligns with the cell’s nucleic acid and hybridizes with the cognitive sites in the hybridization step (Salomon et al., 2015). After hybridization, depending on the chemical modifications in the designed ASOs, different events may occur that change a specific gene’s expression (Crooke et al., 2007).

It seems that some limitations affect the use of ASOs in clinic as therapeutic agents. For instance, ASOs are very sensitive to endonucleases and are susceptible to rapid degradation. Thus, chemical modifications have now been applied to improve their half-life in the cells (Dias and Stein, 2002). Replacement of one non-bridging oxygen atom in the ASO backbone with sulfur is a suitable strategy for enhancing nuclease resistance and binding efficiency to serum proteins (Watanabe et al., 2006). To boost the stability of ASOs and improve their safety and efficacy profiles, chemical modifications at the 2 positions of ribose sugar are utilized, such as 2ʹ-O-methyl (2ʹ-OMe) and 2ʹ-O-methoxyethyl (2ʹ-MOE) (Rinaldi and Wood, 2018). Second-generation ASOs were developed using phosphorothioate backbone and 2ʹ-O modified nucleotides. It was observed that the biological activity and stability of these molecules increased, and the ASOs immuno-stimulatory reduced considerably (Geary et al., 2001; Hamm et al., 2010). Substitution of natural phosphoribose backbone with phosphorodiamidate morpholino oligomers (PMO) is another strategy to increase the resistance of ASOs against nuclease and protease activity (Hudziak et al., 1996). In the PMO, the deoxyribose moiety is replaced by a morpholine ring which is resistant to nucleases (Hudziak et al., 1996).

#### 4.4.1 Antisense Oligonucleotides in Familial Hypercholesterolemia

ASOs are synthesized to regulate the level of some proteins involved in the LDL-c metabolism. To increase the LDL-C uptake in the hepatocytes, second-generation 2′-O-methoxyethyl ASO targeting PCSK9 mRNA was designed and explored. Administration of high doses of this ASO significantly decreased PCSK9 mRNA (92%), increased LDLR protein (2 fold), and reduced LDL-C *in vivo* (Graham et al., 2007). Because of insufficient binding affinity, the development of second-generation ASOs was terminated, and shorter ASOs based on locked nucleic acid technology such as SPC5001 were explored. These ASOs have higher stability, binding affinity, and specificity for PCSK9 mRNA than second-generation and longer oligonucleotides. Administration of ASO reduces the level of PCSK9 mRNA by 60% within 24 h post-injection, and the inhibitory effects remained for more than 16 days (Gupta et al., 2010). In healthy individuals, subcutaneous administration of SPC5001 decreased the levels of PCSK9 mRNA and LDL-C by ∼50 and 25%, respectively (Nishikido and Ray, 2019). Likewise, the SPC5001 ASOs reduced apoB levels and increased apoA1 in the target cells (van Poelgeest et al., 2015). Some side effects are observed following administration of SPC5001, such as transient renal tubular toxicity and injection site reactions, which terminated further clinical development (van Poelgeest et al., 2013). Another anti-PCSK9 ASO was developed with high potency and low toxicity. Administration of aforementioned anti-PCSK9 ASO in mice, twice a week for 6 weeks, showed that the level of PCSK9 mRNA and LDL-C decreased in a dose-dependent manner (Yamamoto et al., 2012) (Figure 1).

Mipomersen is an ASO that binds to ApoB mRNA in the liver and inhibits its translation (Gaudet and Brisson, 2015). In 2010, the randomized, double-blind, placebo-controlled, phase III study using 52 patients with HoFH was conducted. In these patients, 200 mg Mipomersen was administered once a week for 26 weeks, and the results showed that the LDL-C level was reduced by 24.7% (Raal et al., 2010). Likewise, in another study, 104 FH patients were treated with Mipomersen. After 1-year follow-up, the results showed that the mean LDL-c and Lp(a) levels were decreased by 28 and 16.6%, respectively (Duell et al., 2016). According to the results of a 2-year follow-up, the most frequently reported adverse effects were injection-site reactions, elevations of alanine aminotransaminase, and potential hepatic toxicity (Duell et al., 2016).

ISIS-APO(a)Rx is another ASO that targeted the Lp(a) protein level in FH patients to reduce the early onset and severity of coronary artery disease (CAD) (Li et al., 2017). In a phase I clinical trial, subcutaneous administration of 300 mg of ISIS-APO(a)Rx reduced the level of Lp(a) protein and associated oxidized phospholipid (OxPL) by 89 and 93%, respectively. The most common adverse effect associated with this ASO is mild injection-site reactions (Tsimikas et al., 2015).

Another ASO,ANGPTL3-LRx, reduces the level of ANGPTL3 protein, which lowers the risk of cardiovascular disease (Dewey et al., 2017). ANGPTL3 is a secretory protein that modulates plasma lipid levels by inhibiting postprandial lipoprotein lipase activity (Tikka and Jauhiainen, 2016). After 6 weeks of treatment, ANGPTL3-LRx decreases ANGPTL3 protein, TGs, LDL-C, VLDL, apoB, and apoC-III levels by up to 84.5, 63.1, 32.9, 60, 25.7, and 58.8%, respectively (Graham et al., 2017; Table 1).

## 5 The Challenges and Opportunities of RNA-Based Therapeutics for Dyslipidemia

Multiple conventional therapies are available to manage LDL-C levels in FH patients. Statins can up-regulate the LDLR level and reduce the LDL-C in HoFH patients lacking functional LDLR. Therefore, they are not generally effective (Marais et al., 2002). Combination of statins with bile acid sequesters or niacin reduces LDL-C, but potential adverse effects limited their administration (Cuchel et al., 2014). Lomitapide is a small molecule that binds to and inhibits the microsomal triglyceride transfer protein (MTP) in hepatocytes’ endoplasmic reticulum. MTP is an enzyme responsible for the transfer of triglycerides to nascent apoB and the assembly of VLDL and chylomicron (Alonso et al., 2019). MTP inhibition results in post-transcriptional degradation of apoB which leads to lowering the secretion of lipoprotein and then serum cholesterol as well as triglyceride levels (Burnett and Watts, 2007; Jiang et al., 2008). Lomitapide as a small molecule has some adverse effects such as gastrointestinal side effects, accumulation of liver fat, and elevations in alanine aminotransaminase levels (Ito and Watts, 2015). Some monoclonal antibodies were used to treat FH such as Evolocumab and Alirocumab. These anti-PCSK9 monoclonal antibodies were approved by FDA for the treatment of adults. These monoclonal antibodies have some adverse effects including upper respiratory tract infections, gastroenteritis, nasopharyngitis, and influenza (Ito and Watts, 2015). Also, it was shown that the response to evolocumab was dependent on LDLR status because no response was observed in patients with LDLR-negative mutations on both alleles (Raal et al., 2015). Small molecules are other therapeutic agents that can target specific points in LDL-C metabolism. They have some advantages such as ease of administration, low cost, long shelf life, and non-immunogenic response. However, because of some disadvantages including low selectivity, non-tissue specific effects, and severe side effects, their use as medications have faced some limitations (Miyosawa et al., 2015). Mimetic peptides are also new therapeutic molecules designed to mimic peptides of a target protein. They mimic, for example, the EGF-A or EGF-AB binding domains of LDLR or bind to and inhibit the catalytic domain of PCSK9, thereby they can reduce interaction between PCSK9 and LDLR (Craik et al., 2013). Like mAbs and because of limited oral bioavailability, mimetic peptides often require systemic delivery (Bergeron et al., 2015). Vaccination therapy is an alternative strategy for the long-term reduction of LDL-C levels in FH patients. For example, AT04A, a vaccine that mimics the fragments of PCSK9 protein conjugated to a foreign carrier protein (Keyhole limpet hemocyanin), was developed for the management of FH (Landlinger et al., 2017). Besides long-term efficacy of vaccination therapy in FH, potential adverse effects include antibody-dependent cell-mediated cytotoxicity or complement-dependent cytotoxicity, and non-specific cell destruction which have limited their application (Civeira and Jarauta, 2017).

Non-coding RNAs are used in novel theranostic applications to regulate the expression of some genes related to FH diseases. These RNAs can target mRNAs at various levels, both in the same or different pathways. Now, high-throughput technologies are developed to provide valuable information about different ncRNAs, their expression, and the mRNA transcripts which they might regulate (Akhtar et al., 2016). Several ncRNA-based therapies have been developed, and many have been described (Rupaimoole and Slack, 2017). Currently, at least 11 ncRNA-based drugs are approved by the Food and Drug Administration (FDA) or the European Medicines Agency (EMA) to treat different diseases. Moreover, many ncRNAs therapeutics are in clinical phases, but, until now, no ncRNA-based drugs have been considered in clinical settings (Winkle et al., 2021a). MicroRNAs are a large class of non-coding RNAs that play essential roles in regulating gene expression. miRNA-based therapeutics have at least two advantages; first, they are naturally processed and produced in human cells. Second, they act through targeting multiple genes within one pathway and thus have a broader and more specific response (Winkle et al., 2021a). Therefore, clinical application of natural miRNAs could be a promising approach for the treatment of some diseases and may potentially enhance the therapeutic effects as compared with siRNAs or other synthetic RNAs (Rupaimoole and Slack, 2017). Interestingly, using miRNAs analogs could increase the half-life and delivery of miRNAs to the target tissues; for example, in miR-30c analog, the passenger strand of miR-30c was modified which leads to stability enhancement and augment its delivery to liver cells (Yadav et al., 2022). Despite the importance and necessity of ncRNAs in preclinical investigations, their efficient and successful utilization in clinical trials has faced hurdles so far. For instance, there are difficulties in administering miRNAs *in vivo* since they are a subset of non-coding RNA. On one side, negative charges on miRNA and hydrophilic feature restricted its absorption by cells (Kapadia et al., 2020). On the other side, systemic treatment of miRNA and siRNA causes off-target effects (Bartoszewski and Sikorski, 2019). Aside from that, short half-lives and immune responses are critical issues that may limit miRNA clinical trials (Lee et al., 2019). Furthermore, non-coding RNA inhibitors may not have the desired impact on the complete body due to initial accumulation in specific organs (Atri et al., 2019). All in all, the goal of making these ncRNAs more efficient for therapeutic purposes is to reduce toxicity, optimize delivery to the target organ and cell in the first phase, and then recruit for specific cellular functions with high potency and stability (Winkle et al., 2021b).

However, there are some barriers to the use of ncRNA-based therapies in the clinic, including a) the specificity of targets involved in the development of the disease, b) targeted delivery of the drug at the subcellular level, c) stability of ncRNA-based therapeutics, and d) off-target effects (Hueso et al., 2021). For example, several fundamental disadvantages; including scrambling in the biogenesis of the endogenous miR, an evocation of the interferon response, and the off-target effects limit the therapeutic application of siRNA (Navarro et al., 2020). Recently, using improved algorithms for the development of more effective and specific ncRNAs, new chemistries for their stabilizing, and more efficient vehicles for enhancement of their delivery, lead to the development of new and effective ncRNA-based therapeutics.

## 6 Conclusion

Early detection and suitable intervention for FH in order to lower LDL is the standard goal to reduce the associated cardiovascular disease burden. Various non-coding RNAs, including lncRNAs, miRNAs, siRNAs, and ASOs interact with and regulate genes and their transcripts in lipid and cholesterol metabolism. As a therapeutic approach, some lncRNAs such as MIAT, GAS5, RP5-833A20.1, LeXis, Lnc-HC, and DYNLRB2 may serve as crucial regulators of cholesterol homeostasis. Small RNA interference drugs are used to silence disease-causing genes. Inclisiran (ALNPCSsc: ALN-60,212) is an excellent example of siRNAs used to reduce the level of LDL-C in FH. ASOs are another group of ncRNAs synthesized to regulate the level of some proteins involved in the LDL-c metabolism. Mipomersen is a well-known ASO used to inhibit the translation of ApoB mRNA. Also, miRNAs are another class of non-coding RNAs that could be promising tools for the early diagnosis and treatment of FH. Despite the importance and necessity of ncRNAs in preclinical investigations, their efficient and successful utilization in clinical trials has faced some challenges. These data suggest that non-coding RNAs could be promising tools for the early detection and treatment of FH disease, but more research is needed to overcome current challenges.
